# A genomic view of the bacterial family *Endozoicomonadaceae* in marine symbioses

**DOI:** 10.1038/s42003-025-08828-9

**Published:** 2025-10-02

**Authors:** Daniela M. G. da Silva, Rodrigo Costa, Tina Keller-Costa

**Affiliations:** 1 Department of Bioengineering, Instituto Superior Técnico, Lisbon, Portugal; 2 iBB—Institute for Bioengineering and Biosciences and i4HB—Institute for Health and Bioeconomy, Instituto Superior Técnico, Lisbon, Portugal

**Keywords:** Marine microbiology, Microbial ecology, Symbiosis, Environmental microbiology, Bacterial genomics

## Abstract

*Endozoicomonadaceae* bacteria are found in association with marine organisms across ocean ecosystems. Interactions may range from mutualistic to parasitic depending on host species and ecological context. Their genomic repertoire suggests metabolic versatility and capacity for rapid adaptation and transitioning between free-living and host-associated lifestyles. Some lineages, however, undergo genome reduction, are host-specific, and lack cultivability. Here we present an advanced genomic perspective and updated view on the functional diversity of *Endozoicomonadaceae* along the mutualism-parasitism continuum. We discuss their roles in marine symbioses, potential for microbiome engineering, and highlight knowledge gaps of their ecology to be addressed in future research.

## Abstract (web summary)

This review explores the genomic diversity, ecology, and functional potential of the bacterial family *Endozoicomonadaceae* in marine symbioses, highlighting their mutualistic to parasitic spectrum and key gaps to be addressed in future research.

## Introduction

The bacterial family *Endozoicomonadaceae* comprises Gram-negative, rod-shaped, and typically motile species most commonly found in association with benthic marine invertebrates, as well as some fishes^1^. A few members of this family have also been documented in seawater and deep-sea fluids^2,3^. Some *Endozoicomonadaceae* species have been successfully cultured from marine animals, yet there are symbiotic lineages within the family that are either difficult to manipulate in the laboratory or cannot be cultivated at all^4^, resulting in significant knowledge gaps regarding their role in host and ecosystem health. The best-known member of this family is the genus *Endozoicomonas*, the name meaning a monad living inside an animal. Representatives of this genus have been found associated with gastropods^5^, marine sponges^6,7^, corals^8–13^, ascidians^14^, bivalves^15^, and fishes^16^. The interaction of *Endozoicomonas* with corals has received most attention from scientists in recent years, as these relationships appear to be intimate and specialized^17,18^. *Endozoicomonas* spp. are generally considered health indicators and important symbionts for corals due to their high abundance and ubiquity in healthy coral tissues^1^. However, the precise functions of *Endozoicomonas* in the coral holobiont are still not completely unravelled^19^ and much less is known about other genera of the family and the family’s roles in non-coral hosts. This study presents an up-to-date view of the taxonomy, geographic distribution, host range, and putative roles of *Endozoicomonadaceae* in marine symbioses. We provide a contemporary phylogenomic perspective and an extensive functional genomics roadmap to elucidate how symbiosis traits and other functional characteristics are distributed and shared among *Endozoicomonadaceae* clades. By embedding key functional signatures from all publicly available *Endozoicomonadaceae* genomes into their ecological context, we reveal high functional plasticity along the mutualism-parasitism continuum. Finally, we discuss how marine conservation and microbiome engineering could benefit from a mechanistic understanding of the factors driving mutualistic versus pathogenic interactions within the family.

## Phylogeny and taxonomy of the *Endozoicomonadaceae* family

The family *Endozoicomonadaceae* was described in 2018 by Bartz et al.^20^ within the order *Oceanospirillales* (*Gammaproteobacteria, Pseudomonadota*)^21^. It currently comprises three validly published genera—the type genus *Endozoicomonas*^5^, and the genera *Kistimonas*^22^, and *Parendozoicomonas*^20^—and four candidate genera—*Candidatus* Endonucleibacter^23^, *Candidatus* Acestibacter^24^, *Candidatus* Gorgonimonas^4^, and *Candidatus* Sororendozoicomonas^25^. Before the family *Endozoicomonadaceae* was formally described, the genera *Endozoicomonas* and *Kistimonas* were included in the family *Hahellaceae*^8,11,14,22,26^. The phylogenetic analysis conducted by Bartz and colleagues, however, clustered together the genera *Endozoicomonas, Kistimonas*, and *Parendozoicomonas*, as well as *Candidatus* Endonucleibacter bathymodioli^20^, a bacterial parasite that can invade and multiply in the nucleus of bathymodiolin mussels^23^. The family *Endozoicomonadaceae* was thus proposed and is phylogenetically close to the family *Zooshikellaceae*^27^.

Currently, the *Endozoicomonas* genus^5^ comprises 15 species^21^, 11 of which are validly published under the International Code of Nomenclature of Prokaryotes—ICNP^28^: the type species *E. elysicola*^5^ along with *E. montiporae*^10^*, E. euniceicola*^11^*, E. gorgoniicola*^11^*, E. numazuensis*^6^*, E. atrinae*^15^*, E. arenosclerae*^7^*, E. ascidiicola*^14^*, E. acroporae*^8^, *E. coralli*^9^, and *E. lisbonensis*^29^. The remaining species are *Candidatus* E. cretensis^16^, *Candidatus* E. penghunesis^13^, *E. marisrubri*^12^, and *Candidatus* E. endoleachii^30^. The genus *Kistimonas*^22^ currently hosts three valid species: the type species *K. asteriae*^22^, *K. scapharcae*^31^, and *K. alittae*^32^. In 2018, the genus *Parendozoicomonas* was described by Bartz et al. and has so far two validly described species, *P. haliclonae*^20^ and *P. callyspongiae*^33^. Moreover, due to its phenotypic and genotypic similarities with *Parendozoicomonas* strains^33,34^, *Sansalvadorimonas verongulae* was recently proposed for reclassification as *Parendozoicomonas verongulae*^33^. The first uncultured *Endozoicomonadaceae* phylotype was *Candidatus* Endonucleibacter bathymodioli proposed by Zielinski et al. in 2009^23^, followed by *Candidatus* Acestibacter aggregatus, 2010^24^, *Candidatus* Gorgonimonas, 2022, with two species, *Candidatus* G. eunicellae and *Candidatus* G. leptogorgiae^4^, and finally *Candidatus* Sororendozoicomonas aggregata in 2024^25^.

Owing to the recent description of the family *Endozoicomonadaceae*^20^ and the relatively low number of type strains in contrast with many yet unclassified and uncultured phylotypes^35^, there is still some debate about its phylogeny. Some researchers suggest that the family *Endozoicomonadaceae* and the genus *Zooshikella* should belong to the same family and even argue that this family should belong to the order *Pseudomonadales*^27^. To avoid communication gaps between the existing literature and future research, there is a current need to shed light on the classification and evolutionary relationships of species within the *Endozoicomonadaceae* family.

**Fig. 1:**
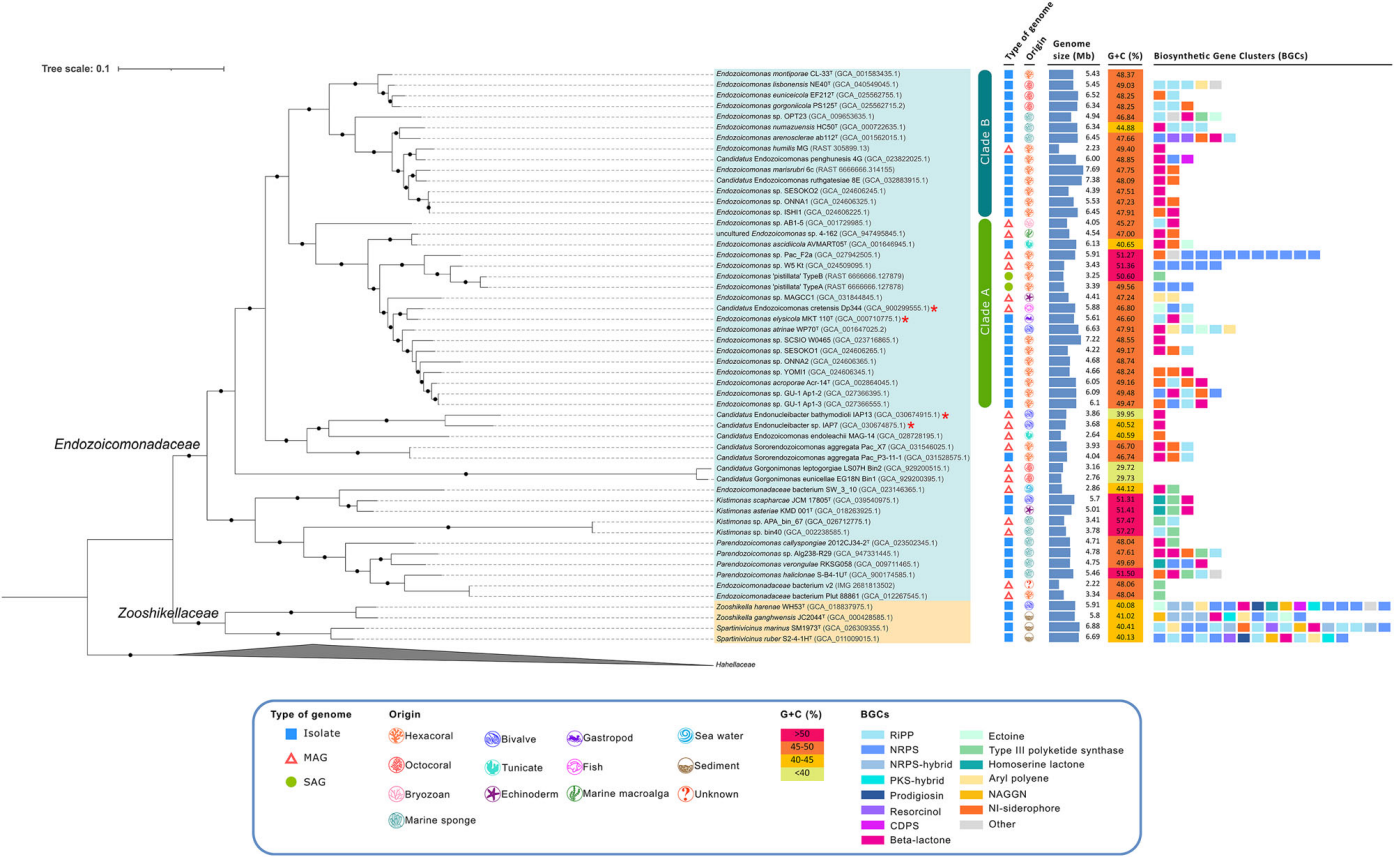
Phylogenomic relationships and genome characteristics of the *Endozoicomonadaceae* family. All genomes, MAGs and SAGs shown here are available on NCBI^126^, IMG^118^ or RAST^117^ (accessed March 2024) and, according to GTDB-tk classification^121^, belong to the family *Endozoicomonadaceae* (*N* = 50) or *Zooshikellaceae* (*N* = 4). All genomes, MAGs, and SAGs shown here have an overall quality score (% Completeness–5$\times$ % Contamination), above 50%^127^. Three genomes of the family *Hahellaceae* serve as an outgroup to root the tree (*Hahella ganghuensis* DSM 17046^T^, *H. chejuensis* KCTC 2396^T^, and *Allohahella marinimesophila* JCM 17555^T^). The phylogenomic tree was calculated using the SpeciesTreeBuilder version 0.1.4 application with the function ‘Insert Set of Genomes into Species Tree’ of the DOE Systems Biology Knowledgebase (KBase) online platform^119^ after annotating the genomes with Prokka^122^ within KBase. Sequences were aligned based on a multiple sequence alignment using a set of 49 core clusters of orthologous groups (COGs) of proteins. The FastTree2 algorithm^123^ was used with the maximum likelihood (ML) method and Jukes–Cantor evolutionary model with a CAT approximation to generate a tree with 1000 repetitions to estimate bootstrap support values^123^. Filled circles on the branches indicate that the respective nodes have a bootstrap value above 70%. Strains highlighted in blue belong to the family *Endozoicomonadaceae*, while those in yellow represent the family *Zooshikellaceae*, as indicated at the corresponding nodes. Known parasitic strains are marked with a red asterisk next to their names and genome accession numbers. Vertical bars next to the tree indicate *Endozoicomonas* clade A and clade B. *Endozoicomonadaceae* and *Zooshikellaceae* origin, genome sizes, GC contents (%) and their secondary metabolite encoding biosynthetic gene clusters (BGCs) are plotted next to the tree. BGCs were annotated with the antiSMASH 7.0 bacterial version online platform^124^. The category ‘Other’ BGCs refers to BGCs that appear only once among all genomes. MAG metagenome assembled genome, SAG single amplified genome, RiPP ribosomally synthesized and post-translationally modified peptide products, NRPS non-ribosomal peptide synthetase, CDPS cyclodipeptide synthase, NAGGN *N*-acetylglutaminylglutamine amide, PKS polyketide synthase. More detailed information on genome quality, general genome features, and BGC annotations can be found in Supplementary Data 1–3.

The phylogenomic view presented here (Fig. 1) includes all available genomes of strains classified as *Endozoicomonadaceae* and thorough metadata documentation (Supplementary Data 1, 2), providing deeper insight into their phylogeny and ecological context. The phylogenomic inference reveals clustering of the genus *Endozoicomonas* into two clades (A and B), as also observed by Pogoreutz et al.^12^. This division likely resulted from complex patterns involved in the establishment of host–symbiont associations, which can be affected by host–symbiont species co-diversification, environmental acquisition of symbionts (e.g. host reproduction strategy), and geographical adaptation^12^, as discussed in the following sections. Moreover, our analysis supports the separation of *Endozoicomonadaceae* from the genera *Zooshikella* and *Spartinivicinus* of the family *Zooshikellaceae*^36^. While all cultivated *Endozoicomonadaceae* strains with available genomes were isolated from marine eukaryotic hosts, most *Zooshikellaceae* members originate from non-host environments. A key difference in coding potential between *Endozoicomonadaceae* and *Zooshikellaceae* genomes is the number and diversity of biosynthetic gene clusters (BGCs) (Fig. 1, Supplementary Data 3). *Endozoicomonadaceae* genomes show a prevalence of BGCs encoding non-ribosomal peptide synthetases (NRPS), beta-lactones, type III polyketide synthases (T3PKS), and siderophores. In contrast, *Zooshikellaceae* genomes contain a greater number of BGCs, often dominated by NRPS and NRPS hybrid clusters. They also feature unique BGCs encoding the osmoprotectant dipeptide *N*-acetylglutaminylglutamine amide (NAGGN) and the pigment prodigiosin, which are absent from *Endozoicomonadaceae* genomes.

### Phenotypic traits of cultured *Endozoicomonadaceae* representatives

All cultured *Endozoicomonadaceae* species are Gram-negative, rod-shaped, and usually show motility (Table 1)^20^. Cells are typically small, measuring <1 μm in diameter and 3–4 μm in length. A notable exception is *Endozoicomonas numazuensis*, which can reach lengths of up to 10 μm. These bacteria are mesophilic neutrophiles that require salts for growth^20^. Their major respiratory quinone is ubiquinone Q-9 and the major polar lipids in their cell envelope are phosphatidylethanolamine, phosphatidylglycerol, phosphatidylserine, and minor amounts of diphosphatidylglycerol^20^. The predominant fatty acids of their cell membranes are mixtures containing palmitoleic acid (summed feature three), palmitic acid (C_16:0_), and often also summed feature eight. The major hydroxyl fatty acid is 3-hydroxydecanoid acid (Table 1). They mostly form small circular colonies on Marine Agar, with colours ranging from translucent to white, beige, or yellow^20^.

**Table 1:** Comparison of phenotypic, physiological and molecular features of the type strains of *Endozoicomonadaceae* species

Species	Endozoicomonas acroporae^8^	Endozoicomonas arenosclerae^7^	Endozoicomonas ascidiicola^14^	Endozoicomonas atrinae^15^	Endozoicomonas coralli^9^	Endozoicomonas elysicola^5^	Endozoicomonas euniceicola^11^	Endozoicomonas gorgoniicola^11^	Endozoicomonas lisbonensis^29^	Endozoicomonas montiporae^10^	Endozoicomonas numazuensis^6^	Kistimonas alittae^32^	Kistimonas asteriae^22^	Kistimonas scapharcae^31^	Parendozoicomonas haliclonae^20^	Parendozoicomonas callyspongiae^33^	Parendozoicomonas verongulae^34^
Year of description	2017	2016	2016	2014	2018	2007	2013	2013	2025	2010	2013	2018	2010	2012	2018	2024	2018
Type strain^a^	Acr-14	ab112	AVMART05	WP70	Acr-12	MKT 110	EF212	PS125	NE40	CL-33	HC50	BGP-2	KMD 001	A36	S-B4-1U	2012CJ34-2	RKSG058
Colony colour	Creamy white	Cream	Beige	Beige	Creamy white	Beige	White	Creamy white	Translucid cream	Beige	Pale creamy white, opaque	Translucent	Light yellow	Beige	Transparent-white, later orange	Transparent, and pale-yellow	Translucent, cream, pale-yellow
Colony morphology	Circular, convex, irregular margins	Circular, convex, entire margin	Circular, convex, smooth surface, entire margins	Circular, convex, entire margins	Circular, convex, irregular margins	Circular, convex, smooth, shiny	Circular, convex	Circular, convex	Circular, convex, smooth, entire margins	Convex, circular, entire margins	Circular, low-convex, undulate margins	Convex, smooth margins	ND	Circular, entire margins	Circular, convex, shiny surface	Circular and convex	Circular, smooth and shiny, entire edges
Colony diameter (mm)	0.8−2.2	ND	0.5−1.0	0.6−1.1	0.5−2.0	4.0−5.0	0.2−0.5	0.5−1.0	0.5–1.5	1.0−2.0	1.5−2.0	0.6−1.0	ND	0.5−1.5	1.0−2.0	ND	ND
Cell length (μm)	2.0−3.0	ND	1.2−11.3	1.2−3.6	1.0−2.2	1.8−2.2	1.7−2.6	1.7−2.5	1.5–3.0	1.0−3.0	3.0−10.0	ND	1.0−1.6	0.6−3.9	2.8−3.2	3.5–4.0	2.0–12.0
Cell diameter (μm)	0.5−0.8	0.5−1.0	0.3−0.7	0.7−1.0	0.5−1.0	0.4−0.6	0.6−0.9	0.4−0.9	0.5–1.0	0.5−0.7	0.4−0.8	ND	0.3−0.5	0.2−0.4	0.6−1.0	0.6–0.9	0.6–1.2
Temperature range for growth (°C) (optimum)	20–30 (30)	12−35 (20−30)	5–27 (23)	15–37 (30)	15−35 (30)	4–37 (25–30)	15–30 (22–30)	15–30 (22–30)	15–37 (28–32)	15–35 (25)	15–37 (25)	10−37 (30−32)	15−30 (25)	10−45 (30−37)	15−37 (25−30)	15–42 (30)	15–42 (30)
pH range for growth (optimum)	5.0–10.0 (7.0)	ND	6.2–8.3 (6.0–7.0)	6.0−9.0 (7.0)	6.0-9.0 (7.0)	6.0−9.0^b^ (7.0−7.5)^b^	7.0–8.0 (8.0)	7.0–9.0 (8.0)	6.0–8.0 (7.0–8.0)	6.0−10.0 (8.0)	5.5−9.0 (7.5−8.0)	6.0−10.0	5.0−10.0	6.0−10.0 (8.0)	6.5−9.0 (7.0−8.0)	6.0–9.0 (7.0)	6.0–9.0 (7.0)
NaCl concentration for growth (%, w/v) (optimum)	1.0−5.0 (2.0)	2.0−4.0 (3.0)	0.5–5.0 (1.0)	1.0−4.0 (2.0)	1.0−4.0 (3.0)	0.5−4.0^b^ (1.0−2.0)^b^	1.0–4.0 (2.0−3.0)	1.0–4.0 (2.0−3.0)	1.0–5.0 (2.0–3.0)	1.0−3.0 (2.0−3.0)	1.25−5.0 (2.0)	1.0 − 4.0	0.5−10.0 (0.5−9.0)	0.0−5.0 (1.0)	0.5−4.0 (1.0−2.0)	0.5–10 (2.0–3.0)	0.5–10.0 (2.0–3.0)
Relation to O_2_	A	A	FAN	A	A	A	FAN	FAN	FAN	A	FAN	FAN	A	FAN	FAN	A	A
Motility	−	+	+	−	−	+	+	+	+	+	−	+	+	+	+	−	+
Reduction of nitrate	+	ND	+	+	+	+	−	−	+	+	+	+	−	+	+	–	−
Catalase	+	ND	+	+	+	+	+	+	+	+	+	+	ND	−	+	+	+
Oxidase	+	ND	+	+	+	+	+	+	+	+	+	+	+	−	+	+	+
*Fatty acids*																	
C_10:0_3–OH	2.8	ND	4.31	2.7	4.3	3.1	3.0	2.4	5.22	2.9	4.9	4.1	−	8.10	5.2	1.4	2.0
C_14:0_	1.1	ND	0.0	2.1	1.4	9.3	13.8	8.5	9.69	8.5	0.9	3.9	0.72	5.22	2.4	6.0	6.8
C_16:0_	13.5	ND	15.7	24.4	14.9	18.9	17.1	17.1	21.95	12.0	20.0	17.6	1.06	17.57	13.5	9.9	16.6
Summed feature 2	1.9	ND	0.91	3.8	3.2	ND	2.8	1.0	4.1	1.5	1.8	ND	−	1.83	0.9	<1.0	2.3
Summed feature 3	45.5	ND	49.6	33.9	36.4	54.5	44.0	49.4	36.1	39.6	31.5	36.6	−	43.90	29.4	75.0	31.0
Summed feature 8	27.2	ND	24.3	28.0	30.4	5.5	15.97	19.45	18.55	32.8	32.9	13.8	−	ND	39.0	4.6	25.6
Respiratory quinones	Q-9, Q-8	ND	Q-9	Q-9, Q-8	Q-9, Q-8	Q-9, Q-8	Q-9	Q-8	Q-9, Q-8	Q-9, Q-8	Q-9, Q-8, MK-9, MK-8	Q-9	Q-9	Q-9	Q-9, Q-8, Q-10	Q-9, Q-8	Q9, Q8

*A* aerobe, *FAN* facultative anaerobe, + positive, −negative, *ND* no data available. Summed feature 2 (C_14:0_3-OH/isoC_16:1_); summed feature 3 (C_16:1_ω7c/C_16:1_ω6c); summed feature 8 (C_18:1_ω7c/C_18:1_ω6c).

^a^Only type strains of valid species according to the ICNP are presented, except for *Parendozoicomonas verongulae* RKSG058, which has not yet been validly published (currently *Sansalvadorimonas verongulae*).

^b^Obtained from Bartz et al. 2018^20^.

### Geographic distribution and preferred biotopes

**Fig. 2:**
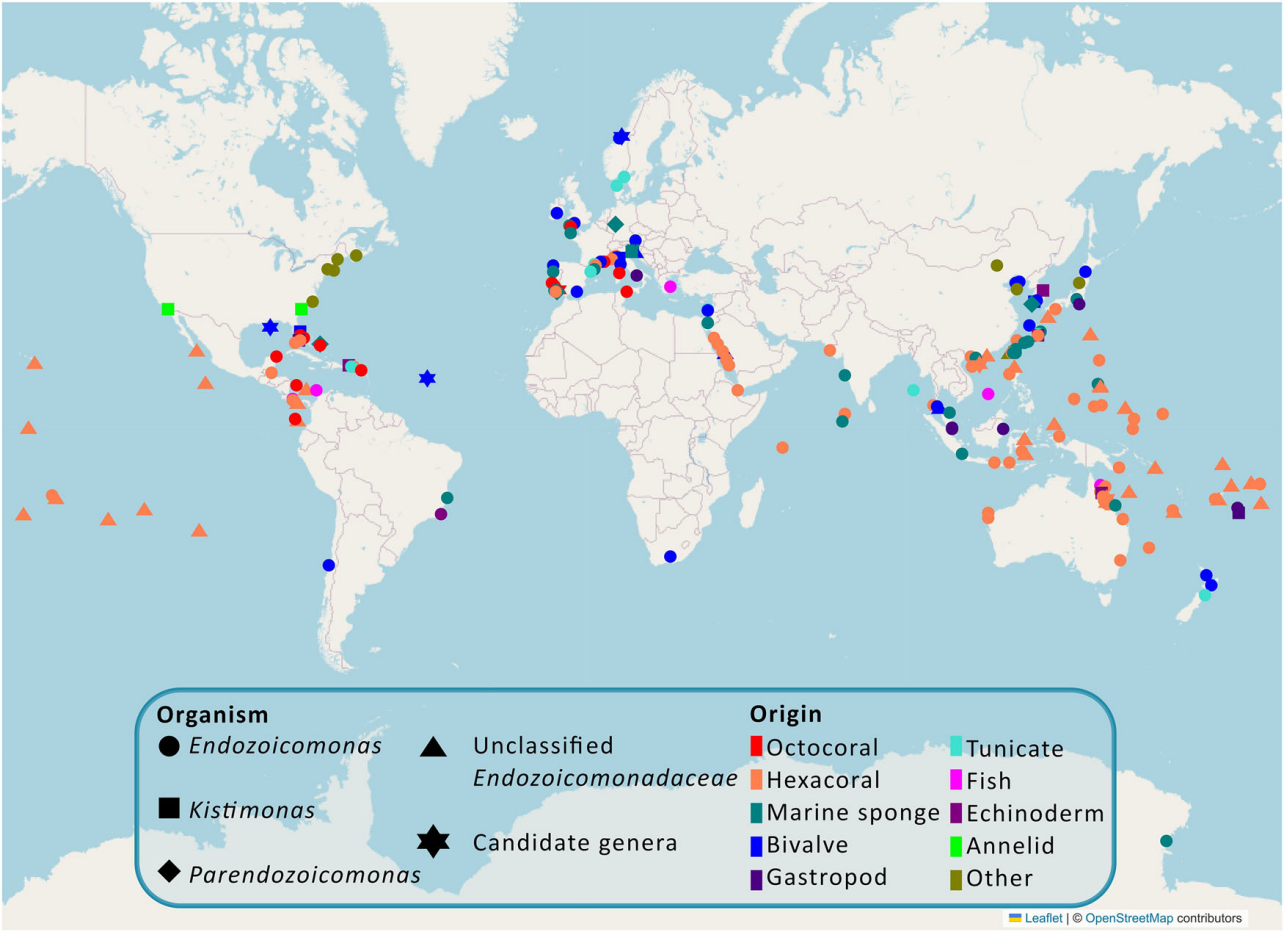
Global distribution and diversity of *Endozoicomonadaceae* associated with diverse host organisms. The map was built with the ‘folium’ library on Python and displays the global distribution of strains attributed to the family *Endozoicomonadaceae* with available geographic information in articles in PubMed^116^ or NCBI BioSample data^128^ as of June 25, 2024. Candidate genera refer to *Candidatus* Acestibacter (Haltenbanken, Norway), *Candidatus* Endonucleibacter (mid-Atlantic Ocean and Gulf of Mexico), *Candidatus* Gorgonimonas (Portugal), and *Candidatus* Sororendozoicomonas (Great Barrier Reef, Australia and East China Sea). The category ‘Other’ refers to hosts that were only reported once for bacteria of this family, including a jellyfish (*Netrostoma setouchianum*), an anemone (*Nematostella vectensis*), a bryozoan (*Bugula neritina*), a marine macroalgae and an insect (*Altica* sp.); or found in non-host environments such as seawater and tidal flats.

*Endozoicomonadaceae* bacteria are found in association with a large variety of marine eukaryotic hosts and are distributed across the world’s oceans, from photic zones to abyssal depths^18^. They are widely present in tropical and temperate coral reefs, ranging from the Great Barrier Reef in Australia to the Caribbean Sea, across the Atlantic, Pacific and Indian Oceans, to the Red and Mediterranean Seas (Fig. 2)^1^. *Kistimonas* representatives have been found in echinoderms^22,37^, bivalves^31^, and annelids^32^ from the Great Barrier Reef, the East China Sea, Korean Sea, in the East and West coasts of the United States, in the Adriatic Sea and the South Pacific Ocean^22,31,32,38^. *Parendozoicomonas haliclonae* was isolated from the marine sponge *Haliclona* sp. cultured in aquarium facilities in Giessen, Germany^20^. *P. callyspongiae*, *P. verungulae*, and *P*. sp. Alg238_R29 were isolated from wild marine sponges—*Callyspongia serpentina*, *Verongula gigantea*, and *Spongia* aff. *officinalis*—collected from the East China Sea, the West Atlantic, and the Northeast Atlantic, respectively^33,34,39^. *Candidatus* Acestibacter was discovered intracellularly, forming aggregates inside gill cells of the bivalve *Acesta excavata* in Haltenbanken cold water reefs in Norway^24^. The genomes of the yet uncultured *Candidatus* Gorgonimonas genus were obtained through metagenomic binning from temperate octocorals of the species *Eunicella gazella*, *E. verrucosa*, and *Leptogorgia sarmentosa*, from the Northeast Atlantic coast of Portugal^4^. *Candidatus* Sororendozoicomonas was found forming cell-associated microbial aggregates (CAMAs) within the mesenterial filaments of *Pocillopora* spp. in the Great Barrier Reef and East China Sea^25^.

*Endozoicomonadaceae* spp. were the most prevalent taxonomic group associated with intracellular microcolonies of bacteria (IMC) in economically important marine mollusc species from Europe, Africa, Asia, Australia, and South America^40^. Cano et al.^40^ argue that attributing the wide geographical distribution of *Endozoicomonadaceae* in bivalves solely to the international trade of infected seedstock is insufficient, considering historical records of IMC infections in bivalves. Instead, the presence of *Endozoicomonadaceae* in bivalves may rather result from the adaptation, evolution, and natural distribution of this bacterial group^40^.

While there are plenty of studies reporting *Endozoicomonadaceae* as part of the microbiome of marine invertebrates in tropical and temperate regions, few studies have looked at polar regions and the deep sea, wherefore less is known about *Endozoicomonadaceae* prevalence in cold environments. The Antarctic sponge *Mycale (Oxymycale) acerata*, collected in Tethys Bay, had *Endozoicomonas* sequences in its metagenome^41^. Although *Endozoicomonas* spp. were extremely rare or even absent in the deep-sea corals *Anthothela* spp., *Desmophyllum pertusum, Paramuricea placomus*, and *Primnoa* spp. at depths below 400 m^42^, *Endozoicomonas* spp. were found to be dominant in *Acanthogorgia* spp. and in site-specific *Desmophyllum pertusum* at depths below 1000 m and around 4 °C^43^. Overall, this shows that the family has a cosmopolitan geographic range from polar over temperate to tropical regions (Fig. 2) and can be found at greater depths than those that have been more extensively explored.

## *Endozoicomonadaceae* in host–microbe interactions: from mutualism to parasitism

Host–microbe interactions may change and develop depending on the ecological context and can be represented in a flexible gradient known as the ‘mutualism parasitism continuum’^44^. Species in the *Endozoicomonadaceae* family are generally viewed as potential bacterial symbionts in marine ecosystems, and several beneficial and harmful functions have been proposed for the group^1^. The enrichment of genes associated with nutrient acquisition, as well as carbon, nitrogen, and sulfur utilization, protein secretion, and the synthesis of amino acids and vitamins in their genomes suggests aptitude for interacting with host metabolism. These features may support a mutualistic lifestyle by facilitating nutrient exchange or provisioning beneficial metabolites to the host^1^. It has also been suggested that *Endozoicomonadaceae* bacteria may play a role in the regulation of the overall microbiome structure of the coral host, either by direct competition or by producing antimicrobial compounds^45–47^.

*Endozoicomonas* spp. are believed to benefit sponge health^48^ with potential roles in antibiotic production^47^, carbohydrate fermentation, and nitrate reduction^6^. However, this hypothesis is based mostly on genomic inferences, and experimental confirmation of the roles of *Endozoicomonas* spp. in marine sponges is still lacking, a gap that should be addressed in future research. Notably, all *Parendozoicomonas* species proposed so far, and genome-sequenced isolates have been derived from marine sponges^20,33,34,39^. However, the limited number of cultured representatives and accurately identified metagenome sequences make it difficult to determine whether this taxon is truly sponge-specific.

The presence and significance of *Endozoicomonas* species in host–microbe associations have been particularly notable in corals. Using 16S rRNA gene amplicon sequencing, these bacteria have been documented from various coral species, often representing a substantial portion of the coral bacterial communities—sometimes reaching over 96% in relative abundance^49^. This prevalence suggests that *Endozoicomonas* are important for the coral holobiont, although their role is not fully understood. Their high relative abundance in corals is typically linked to a healthy, eubiotic state, while their abundance often decreases in the diseased state of the host^50,51^. Several studies have relied on metagenomics approaches to assess coral bacterial community composition and analyse *Endozoicomonadaceae* spp. relative abundances in corals impacted by different factors. In *Acropora millepora, Endozoicomonas* spp. were found to be less abundant when the seawater pH was low^46^, during increased temperatures and subsequent bleaching^52^, and *Porites astreoides* harboured fewer *Endozoicomonas* reads in lesioned colonies when compared to the non-lesioned ones^53^. However, there are cases where *Endozoicomonas* phylotypes have been found to be more abundant in corals under stress conditions, including coral community loss^51^, microbial community shifts, increased abundance of pathogenic microorganisms^51,54^, and even coral bleaching and tissue sloughing^55^. For example, *Endozoicomonas* phylotypes were more abundant in *Pocillopora verrucosa* and *Acropora hemprichii* at sites impacted by municipal wastewater^51^, in *Porites* spp. under low pH^56^, and in corals in French Polynesia after warm summer months^54^. Given this spectrum of (at times conflicting) observations, changes in *Endozoicomonadaceae* relative abundances detected through amplicon or metagenome sequencing alone are insufficient to infer a beneficial role of this family in corals. Controlled mesocosm experiments that test the effect of *Endozoicomonadaceae* bacteria on corals are urgently needed to respond to this timely query in coral symbiosis research.

Members of the *Endozoicomonadaceae* family can adapt to a wide range of hosts and environments. While they are considered putative mutualists in corals, sponges, and tunicates, they can be pathogens and indicators of dysbiosis in bivalves, echinoderms, and fishes^1^. *Kistimonas scapharcae* was isolated from a dead ark clam when researchers were trying to characterize possible pathogenic bacteria following a mass mortality event within cage-cultured ark clams^31^. Sequences of *Endozoicomonadaceae-*like organisms represented the most abundant bacterial group associated with IMCs, intracellular microcolonies of bacteria that can develop to large extracellular cysts infecting the gill and digestive gland of marine mollusc species^40^. The presence of IMCs is, however, not always correlated with pathology, indicating that the onset of the disease and the severity of the lesions could be associated with environmental factors, host susceptibility, and the *Endozoicomonadaceae* lineage present within the mollusc tissues^40^. IMC-forming lineages were identified to belong to the genera *Endozoicomonas, Parendozoicomonas*, and *Candidatus* Endonucleibacter, the latter being associated with severe lesions^40^. *Candidatus* Endonucleibacter parasitism in other bivalves has also been observed. A single bacterial cell can infect the nucleus of bathymodiolin mussels, where it reproduces rapidly, causing the mussel’s nucleus to swell and eventually burst, releasing numerous bacteria into the seawater^23^. Remarkably, this intracellular parasite can prevent host cell death by producing inhibitors of apoptosis (IAPs) that bind to caspases, allowing it to replicate in high numbers while acquiring the necessary nutrients and energy from the host^57^.

In fishes, some *Endozoicomonas* strains have been found to be pathogens, causing epitheliocystis, a serious infectious bacterial disease characterized by cysts in the skin and gills of juvenile and older fishes^16,58^. These include *E. elysicola*, responsible for epitheliocystis in cobia larvae^58^, and *Candidatus* Endozoicomonas cretensis, causing the formation of cysts in sharpsnout seabream larvae^16^. Such infections affect both wild and captive fish, posing a significant challenge to aquaculture^16,58^. Moreover, *E. elysicola* was suggested to play a role in the development of proliferative gill inflammation in salmon^59^. Captive crown-of-thorns sea stars, well-known coral-eating echinoderms, had *Endozoicomonas* and *Kistimonas* detected in all their tissues during a disease event, with *Kistimonas* present in all diseased individuals and significantly associated with the affected tube feet^37^.

These studies suggest that certain *Endozoicomonadaceae* species may opportunistically transition between different symbiotic relationships depending on the ecological context^60^. However, to date, there is no experimental evidence supporting the notion that a specific *Endozoicomonadaceae* species can simultaneously act as a mutualist in one host species and a pathogen in another.

## Transmission mode, phylosymbiosis, and frequency of occurrence of *Endozoicomonadaceae*

Due to their consistent association with a wide range of coral species around the globe (Fig. 2), *Endozoicomonadaceae* are often considered a ‘core symbiont’ of hexacorals, and have also been found in healthy octocorals, particularly gorgonians, in very high relative abundance^17,49,50^. In addition, the same *Endozoicomonadaceae* phylotype can be present in different coral hosts with distinct relative abundances that may be influenced by the host lineage and environmental conditions^45^. Phylosymbiosis, the correlation between microbial community structure and host phylogeny, has been observed across several coral taxa^18,61–63^. Mediterranean octocorals, including *Paramuricea clavata* and *Eunicella* spp., harbour species-specific *Endozoicomonadaceae* phylotypes^61^. Similarly, other Atlantic and Mediterranean octocorals—*E. labiata*^62^, *E. singularis*, *E. cavolini*, and *C. rubrum*^63^—are dominated by octocoral-specific *Endozoicomonadaceae* lineages^62,63^. This reveals that some corals share an evolutionary history shaped by a long-term partnership and potential coadaptation with their *Endozoicomonadaceae* symbionts^64^.

A coral’s reproductive strategy influences the transmission type and biogeographic distribution of *Endozoicomonas* symbionts. The hexacoral *Pocillopora verrucosa* is a broadcast spawner (i.e., eggs and sperm are released into the water for external fertilization and larval development), which acquires most prokaryotes from the surrounding seawater, permitting greater symbiont variability and weak geographic clustering of the microbiome^17^. Indeed, *P. verrucosa* colonies from various reefs harbour similar *Endozoicomonas* phylotypes without obvious geographic clustering^17^. *Stylophora pistillata*, of the same coral family, however, is a brooding coral (i.e., it has internal fertilization and releases settlement-competent larvae), and symbionts can be transferred vertically to the offspring, resulting in a tighter control of the microbiome and strong geographic structuring. Accordingly, *S. pistillata* from different reefs is associated with geographically distinct *Endozoicomonas* phylotypes and showed strong geographic structuring^17^. In *Pocillopora acuta*, a coral that can reproduce either by broadcast spawning or brooding, *Candidatus* Sororendozoicomonas was found in both adults and larval offspring, suggesting vertical transmission^25^. Congruently, the study also found a geographic structuring of *Endozoicomonadaceae* associations, since *Candidatus* Sororendozoicomonas was not detected in other *P. acuta* populations in the Great Barrier Reef^25^.

In the marine sponge *Monanchora arbuscula*, *Endozoicomonadaceae* was the third most abundant microbial family, comprising 9.5% of the sponge’s total prokaryotic community, as assessed through 16S rRNA gene amplicon sequencing^65^. In ascidians, *Endozoicomonas* are considered specific yet facultative symbionts, existing as commensals living off the mucus continuously secreted into the pharynx^66^. Their presence as extracellular microcolonies in the pharyngeal epithelium of ascidians, along with their detection in surrounding seawater, suggests that these symbionts may be mostly horizontally acquired^66^. Additionally, *Endozoicomonas* have been proposed as core symbionts in the sea squirt *Ciona intestinalis*, as they are consistently detected in the gut microbiome of individuals from geographically distant populations^67^.

It is noteworthy, however, that similar *Endozoicomonadaceae* species have been reported across host species belonging to distinct animal phyla. For instance, sequences with high similarity to the species *E. elysicola*, originally isolated from the sea slug *Elysia ornata*^5^, have been detected in cobia (*Rachycentron canadum*) larvae (99% 16S rRNA gene similarity)^58^ and in *Irciniidae* sponges (98% similarity)^68^. Additionally, 16S rRNA gene sequences with 97.5% similarity to *E. montiporae*, originally isolated from the coral *Montipora aequituberculata*, have been identified in the endemic marine sponge *Arenosclera brasiliensis*^47^. Conversely, as noted above, several *Parendozoicomonas* species have, so far, only been described from marine sponges, *Candidatus* Gorgonimonas exclusively from gorgonian corals, and *Candidatus* Endonucleibacter exclusively from bivalves. Altogether, these observations reinforce broader trends of phylosymbiosis or speciation within certain lineages of the *Endozoicomonadaceae* family, contrasting with a more promiscuous host range observed in other *Endozoicomonadaceae* species.

## Organization and localization inside the host

*Endozoicomonadaceae* spp. occupy different organs and niches in diverse animals. *E. elysicola* was first isolated from the digestive tract of the sea slug *Elysia ornata*^5^*, E. ascidiicola* from the pharynx of the tunicate *Ascidiella* sp.^14^, and *E. atrinae* from the intestinal tract of *Atrina pectinata* clam^15^. In corals, several *Endozoicomonas* spp. have been detected in tissues^1,69,70^ and mucus^53,71,72^ using fluorescence in situ hybridization (FISH) with specific probes coupled to imaging techniques as well as 16S rRNA gene amplicon sequencing methods. *Endozoicomonas* spp. are known to form cyst-like structures or bacterial aggregates, also known as CAMAs, inside their coral hosts^60,69^. These structures can serve as phosphorus reservoirs by participating in phosphate sequestration and cycling in the coral holobiont^73^, potentially facilitating broader nutrient and metabolite exchange between the host and other holobiont partners^73^. CAMAs formed by *Endozoicomonas* spp. in corals can be either intra- or extracellular^69^, and are often found near the coral’s Symbiodiniaceae^70^. These structures have been primarily observed using FISH and have also been excised from tissues by laser capture microdissection (LCM) for DNA metabarcoding^69^. Although the assembly of these structures is relevant to the holobiont, as they can function as centres of protein transformation and production^1^, CAMAs remain largely uncharacterized^69,73^. Tfp pili are likely responsible for the formation of such aggregates and attachment and movement of *Endozoicomonadaceae* symbionts inside the host^4^. *E. montiporae* forms CAMA-like structures in vitro when supplied with dimethylsulfoniopropionate (DMSP)^74^, indicating that corals or their Symbiodiniaceae may produce metabolites that trigger CAMA formation. In *Porites astreoides, Endozoicomonas* spp. were detected in the ‘coral ecosphere’, a distinct environment surrounding individual coral colonies that acts as an interaction zone between the coral surface and the seawater^75^. This may result from the shedding of coral mucus or tissues, or it could indicate that *Endozoicomonas* cells persist in the seawater and are attracted to the coral surface^75^.

Epitheliocystis in fish larvae, caused by *E. elysicola* and *Candidatus* Endozoicomonas cretensis, is characterized by densely packed bacterial cysts in the periphery of the fins, and skin and gill epithelia^16,58^. Many *Endozoicomonadaceae* 16S rRNA gene sequences were associated with IMCs infecting the gills and digestive glands of various molluscs, sometimes forming extracellular cysts^40^. These sequences were identified as belonging to *Endozoicomonas*, *Parendozoicomonas*, and *Candidatus* Endonucleibacter^40^. Moreover, *Candidatus* Endonucleibacter bathymodioli was detected in the bivalve *Bathymodiolus puteoserpentis* and other bathymodiolin species^23^, occurring in the cell nuclei of the gill, gut, digestive gland, labial palp, mantle, and foot tissues. *Candidatus* Acestibacter aggregatus was also found forming cyst-like aggregations in *Acesta* clams^24,76^. Taken together, *Endozoicomonadaceae* tend to organize in aggregates within host tissues, which, in corals, may enhance holobiont fitness through metabolic interactions and nutrient exchange^73^. However, in bivalves and fishes, these cyst-like structures are often linked to host illness^23,58^.

## General genome features involved in symbiosis of the *Endozoicomonadaceae* family

*Endozoicomonadaceae* genomes vary in size and are often quite large, lacking the streamlining typically associated with obligate endosymbiotic lifestyles. This suggests that they are not host-restricted and likely have free-living stages before host invasion, particularly when moving between hosts, which requires the maintenance of a complete gene repertoire^60^. The genome size of *Endozoicomonadaceae* spp. varies between 4.22 and 7.69 Mb among culturable strains (Fig. 1) but is smaller among metagenome-assembled genomes (MAGs) and single-amplified genomes (SAGs), ranging from 2.22 to 5.91 Mb^77^. The genome sizes are similar to those of other close relatives within the order *Oceanospirillales*. For example, genomes of cultured representatives from the family *Zooshikellaceae* are of comparable size, ranging from 5.8 to 6.88 Mb (Fig. 1). Nevertheless, studies on uncultured *Endozoicomonadaceae* lineages have identified more streamlined genomes. For example, *Candidatus* Gorgonimonas MAGs are relatively small (2.8–3.2 Mb)^4^, and similar genome sizes were observed in other uncultivated *Endozoicomonadaceae* lineages, including unclassified *Endozoicomonadaceae* and *Candidatus* Endonucleibacter (Fig. 1). This suggests that *Endozoicomonadaceae* genomes can be streamlined in highly specific, uncultivated host-associated clades, while cultivated *Endozoicomonas* strains do not exhibit genome streamlining and likely follow a facultative symbiotic lifestyle, as supported by Shao et al.^78^.

The GC-content of *Endozoicomonas* and *Parendozoicomonas* genomes is similar, presenting 40.7–51.4% and 47.6–51.5% in range, respectively, whereas *Kistimonas* spp. have a higher GC-content, ranging from 51.3 to 57.5% (Fig. 1)^77^. However, the GC-content is considerably lower in certain unculturable lineages, e.g., 29.7% in *Candidatus* Gorgonimonas^77^.

**Fig. 3:**
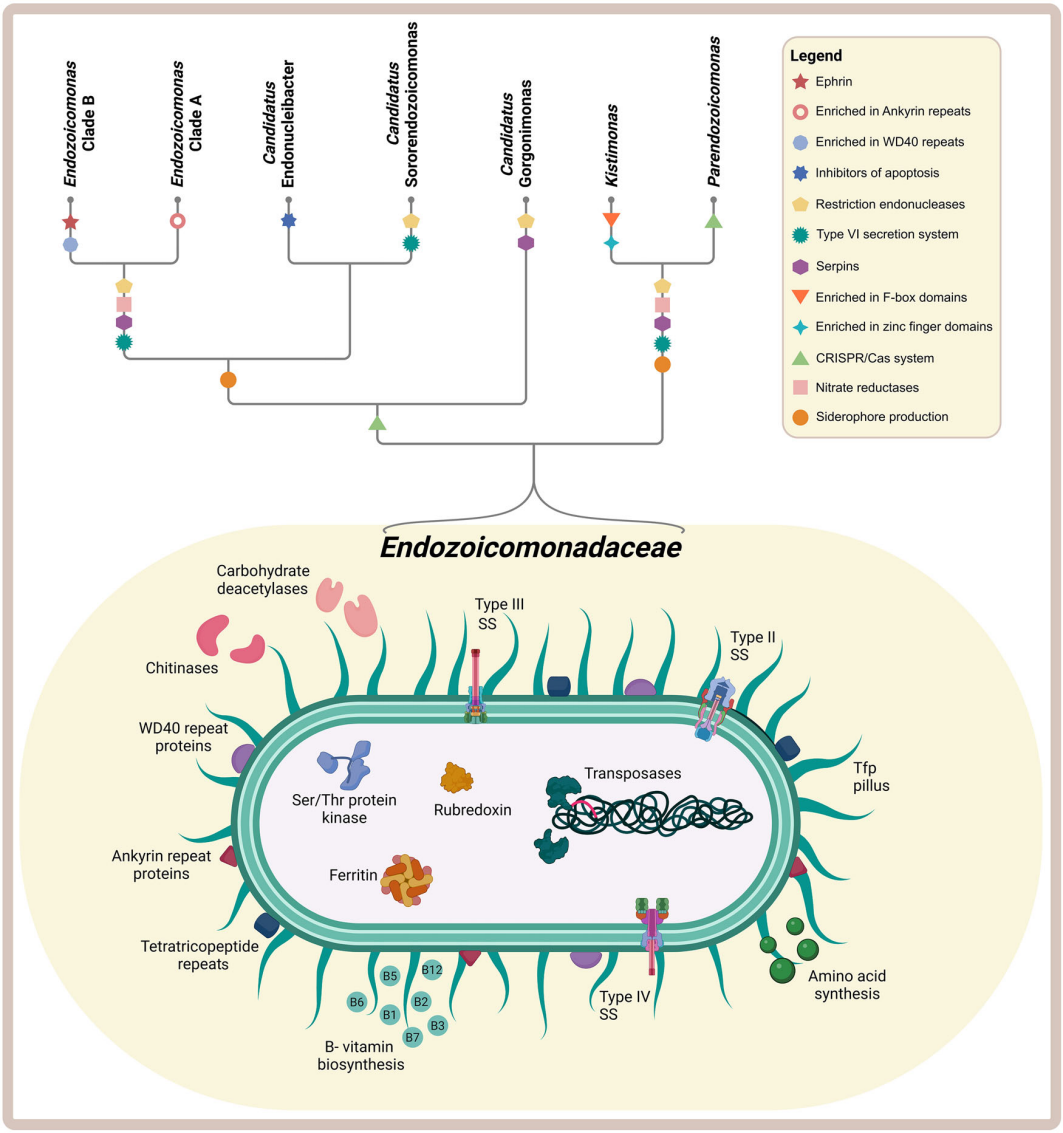
Schematic drawing of common versus clade-specific genome functions within the *Endozoicomonadaceae* family. The schematic bacterial cell drawing depicts the shared functional features across multiple lineages of this bacterial family, while the cladogram represents the relationships among *Endozoicomonadaceae* genera, together with clade-specific functions. The figure summarizes the findings from Clusters of Orthologous Groups (COGs) and Protein family (Pfam) annotations of *Endozoicomonadaceae* genomes (*N* = 50; see Supplementary Data 4–6). Part of this figure was created in BioRender.com: Silva, D., Keller-Costa, T. (2025) https://BioRender.com/yz7gmpl and further edited in Inkscape.

All *Endozoicomonadaceae* genomes present a high number and diversity of transposase-encoding genes (Fig. 3 and Supplementary Data 4–6), which may be responsible for the divergence of genotypes and related to the transition from a free-living stage to a host-associated lifestyle^60^. The enrichment in transposase genes further suggests that *Endozoicomonadaceae* can rapidly rearrange their genomes to adapt to different host niches or environmental conditions, and to transition between mutualistic and parasitic phenotypes^1,18,60^. The genomes of many *Endozoicomonadaceae* species comprise genes related to symbiosis establishment, including type IV pili, WD40, ankyrin, and tetratricopeptide repeats, and type II, III, and VI secretion systems (Fig. 3)^12,69,73,74^. *E. montiporae* has genes coding for *N-*deglycosylation—enzymes hypothesized to help it penetrate the coral mucus—and other genes involved in initiating internalization and evasion of the host immune response^79^. *Endozoicomonas* sp. OPT23 from the marine sponge *Ophlitaspongia papilla* shows adaptive genomic signatures that might favour its symbiotic lifestyle within the sponge host, among them the type III secretion system and effector proteins such as YopH, IpgD, HopI1, HopJ1, and PipB2, able to act as virulence factors to enhance bacterial invasion and dissemination^80–82^. Eukaryotic-like proteins (ELPs), such as ankyrin repeats, tetratricopeptide repeats, and Sel1 repeats, are also present, presumably allowing the bacterium to evade the host immune response and persist inside host cells^12,82^.

Our recent comparative genomics survey^77^ revealed that ELPs are highly abundant in the two major *Endozoicomonas* clades A and B, and that shifts in abundance of ELP encoding genes underly the split of these clades into two functional groups (illustrated in Fig. 3, see also Supplementary Data 4–6)^77^. While clade A is enriched in ankyrin repeats and likely presents a higher diversity of ankyrins^12^, WD40 repeats are more abundant in clade B. Thus, ankyrin and WD-40 eukaryotic-like repeat proteins are genomic signatures of *Endozoicomonas* genomes, with both motifs displaying distinguishing enrichments across the two major clades within this genus. Our comparative genomics analyses further showed that *Kistimonas* genomes are enriched in zinc finger proteins and F-box domains. Like ankyrin and WD40 repeat proteins, bacterial zinc finger domains play, amongst others, roles in host–microbial interactions, as well as in transcription regulation of both prokaryotic and eukaryotic promoters^83,84^. F-box domains are typically associated with protein interaction domains such as ELPs and, in *Legionella*, have been shown to play roles in intracellular proliferation within the host^85^. Altogether, this suggests that the abundance and distribution of ELPs shape the genome evolution and adaptive features of *Endozoicomonadaceae* spp. within host-associated environments. However, the precise mechanisms through which ELPs contribute to the establishment of *Endozoicomonadaceae* symbionts within their host are far from being fully understood. Transposases are further responsible for the dissimilarities between *Endozoicomonas* clades, with some types being more enriched in clade A and others more in clade B^77^. Moreover, ephrins are only present in genomes of *Endozoicomonas* clade B (Fig. 3 and Supplementary Data 5, 6), suggesting that ephrins display clade- or species-specific patterns of occurrence^12^. Presumably, the mechanisms of host–symbiont recognition, internalization, and persistence within the host, along with rapid genome rearrangement, are key factors that distinguish clade A from clade B.

In the uncultured genus *Candidatus* Gorgonimonas associated with temperate octocorals, several features that could facilitate colonization by interfering with host immune responses were detected, including ankyrin repeats, as well as genes associated to type III secretion system, serine/threonine protein kinases, serine protease inhibitors, and effector protein YejM^4^. Moreover, *Candidatus* Gorgonimonas MAGs encode tfp pili, structures required in many functions, including bacterial aggregation, adhesion, and movement on solid surfaces such as host cells^4^.

Exposure to coral extracts was shown to stimulate differential gene expression in *Endozoicomonas*, suggesting that the host’s chemosphere may facilitate symbiosis establishment^12^. The study on *E. marisrubri* 6c suggests that *Endozoicomonas* symbionts find suitable environments via flagellar motility and chemotaxis and, upon encountering a coral host, they downregulate the expression of flagellar assembly genes, initiate internalization via phagocytosis through ephrin receptor binding by EFNB2, upregulate the expression of ankyrin repeats to induce phagosomal arrest and of serpins that interfere with digestion by inhibiting host proteases and peptidases^12^. Following internalization, the bacteria proliferate and form aggregates in the host tissues, for example, near the photosynthetic microalgae symbionts Symbiodiniaceae^12,70^.

Under stressful conditions, benign *Endozoicomonadaceae* symbionts may, however, shift to a pathogenic phenotype, as many of the genome features mentioned above are not exclusive to either mutualistic or parasitic lifestyles^23^. Both relationships utilize similar molecular mechanisms with shared evolutionary histories, making the resulting symbiosis dependent on the ecological context.

## Ecogenomic functions and proposed roles of *Endozoicomonadaceae*

**Fig. 4:**
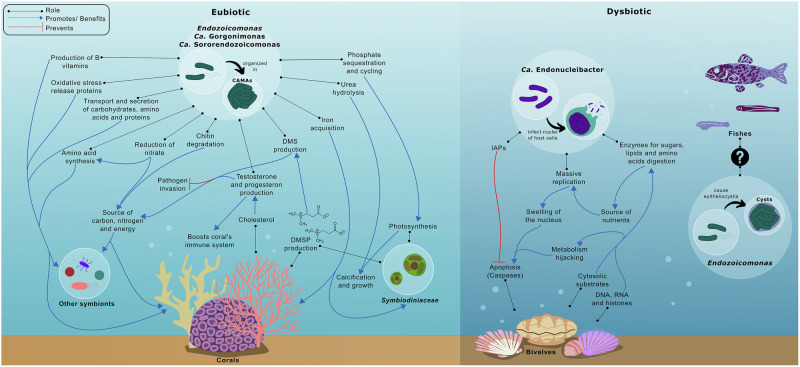
Proposed roles and interactions of *Endozoicomonadaceae* in marine invertebrates, comparing their functions in corals under eubiosis with those in bivalves and fish under dysbiosis. Note that most of these proposed functions are based on genomic studies and/or in vitro evidence, while experimental in vivo verification in the animal host remains largely lacking and represents a key frontier for future research. CAMAs cell-associated microbial aggregates, DMS dimethylsulfide, DMSP dimethylsulfoniopropionate, IAPs inhibitors of apoptosis. This figure was created in Inkscape.

Despite the widespread association between *Endozoicomonadaceae* bacteria and diverse marine hosts, their functions are still under debate due to a lack of experimental in vivo verification. Several roles have been proposed, mainly by means of genomics and some in vitro assays, which can affect the host directly, influence the interactions among the host microbiota, and the microbe-host associations, resulting in an intricate web of interactions affecting the entire holobiont^60^. Figure 4 provides a synthesis of these possible roles of *Endozoicomonadaceae* in ecosystem functioning and health and disease states of their hosts.

The relationship between Symbiodiniaceae and *Endozoicomonas* symbionts is assumed to be a symbiotic partnership that contributes to coral fitness^60^. Neave and colleagues suggested that *Endozoicomonas* can benefit the host by consuming degrading algal cells, since their phosphoenolpyruvate-dependent sugar phosphotransferase system (PTS) mostly encodes for lactose and cellobiose-specific subunits, the latter deriving from cellulose, a major constituent of algal cells^60^. The PTS system may also be involved in chemotaxis, in search for optimal microniches within their host, or the detection of quorum-sensing molecules, which could provide an essential communication route when *Endozoicomonadaceae* form aggregates inside the host^60^. Chemotaxis-related genes are present in several *Endozoicomonadaceae* genomes (Supplementary Data 4–6), which supports the idea that these bacteria can detect and move towards chemical cues^17^. Curiously, only three *Endozoicomonadaceae* genomes—*K. scapharcae*, *K. asteriae*, and *P. verongulae*—carry homoserine lactone (HSL) BGCs involved in quorum-sensing (Fig. 1 and Supplementary Data 3). Most *Endozoicomonadaceae* species possess acyl-homoserine lactone acylases, which biologically inactivate acyl-HSLs. These acylases may have evolved to help bacteria dominate a specific niche by quenching the quorum-sensing signals of competitors^86^.

### Amino acids and B-vitamins production

The genomes of many *Endozoicomonadaceae* species comprise genes involved in the biosynthesis of the vitamins thiamine (B_1_), riboflavin (B_2_), biotin (B_7_), and pyridoxine (B_6_), and pathways for the nine essential amino acids (illustrated in Fig. 4)^60^. The genome of *Candidatus* Acestibacter aggregatus was found to possess complete pathways for the synthesis of eleven amino acids and six B-vitamins, and a minimal chitinolytic machinery, involved in the degradation of polysaccharides that may assist host nutrition^76^. *E. elysicola, E. numazuensis*, and *E. montiporae* contained complete pathways for amino acid synthesis, including alanine, aspartate, cysteine, glycine, homocysteine, homoserine, serine, and the essential amino acids leucine, lysine, methionine, and threonine^60^.

### Phosphorous and iron cycling

*Endozoicomonas* aggregates were found to be enriched in phosphorus, suggesting that they may also play a role in sequestering and cycling phosphate^73^. High levels of phosphate can impede Symbiodiniaceae photosynthesis and consequently decrease the calcification process and the growth of the coral. Hence, phosphate accumulation within these aggregates may represent a buffering mechanism to modulate phosphate abundance within the coral tissues, allowing proper photosynthesis by the endosymbiotic algae and optimizing coral growth (illustrated in Fig. 4)^73^. Genomic data supported the possible role in phosphate cycling as the corresponding *Endozoicomonas* MAGs found in *S. pistillata* presented genes associated with polyphosphate synthesis, uptake, and release^73^.

In marine environments, iron is a limiting nutrient that can hamper primary productivity in corals^4^. Siderophores are low-molecular-weight iron chelators secreted by bacteria to acquire iron in iron-limited environments. Siderophore BGCs were detected in many *Endozoicomonas* strains and in *Candidatus* Sororendozoicomonas, indicating their putative role in iron acquisition under iron starvation (Fig. 1 and Supplementary Data 3)^4^. Iron-chelating activity was also demonstrated experimentally, e.g., in *E. lisbonensis*^29^. However, not all *Endozoicomonadaceae* harbour siderophore BGCs, perhaps because these secondary metabolites are unique and not yet recognized by current annotation platforms, or because they use alternative pathways, such as haem transporters, to acquire iron^87^. Ferrous iron (Fe^2+^) is usually available in anaerobic environments and some *Endozoicomonadaceae* genomes, e.g., *Candidatus* Gorgonimonas, encode ferrous iron uptake systems, underpinning their facultatively anaerobic lifestyle^4^. Most *Endozoicomonadaceae* genomes also possess ferritin encoding genes for iron storage (Fig. 3 and Supplementary Data 4–6), an important mechanism when facing periods of iron depletion^4^. These versatile iron-sequestration mechanisms may represent a key role for *Endozoicomonadaceae* in symbiosis, as they may benefit the host through iron provisioning or influence bacterial community structuring and pathogen control (Fig. 4). Nevertheless, these benefits remain speculative and require experimental validation. After all, the ability to efficiently acquire iron may render *Endozoicomonadaceae* less susceptible to host-imposed iron limitation, a common strategy for controlling microbial populations. Unravelling the dynamics of iron availability in *Endozoicomonadaceae*–host interactions represents yet another frontier for future research.

### Carbon, nitrogen, and sulfur turnover

*Endozoicomonadaceae* bacteria presumably contribute to coral holobiont metabolism through cycling of carbon, nitrogen, and sulfur^46,60,74,87^. Although they are not able to fix nitrogen, the reduction of nitrate to nitrite is frequently reported^20,29,32,60^ (see also Table 1), and E. *coralli* is able to reduce nitrate all the way to nitrogen^9^. Our comparative genomics analysis (see Supplementary Data 4 and 5) indicates that nitrogen cycling pathways in *Endozoicomonadaceae* genomes are predominantly dissimilatory. The identified nitrate reductases primarily correspond to the periplasmic nitrate reductase (Nap) system, which exhibits high affinity for nitrate and is typically more efficient under aerobic or microaerophilic oxygen conditions^88^. In one, yet uncultured *Endozoicomonadaceae*, we also detected the membrane-bound respiratory nitrate reductase (Nar) complex, which facilitates dissimilatory nitrate reduction during anaerobic respiration^88^. Nar-type nitrate reductases appear to be common in the closely related family *Zooshikellaceae*. Compared to nitrate, fumarate may represent a more common terminal electron acceptor under anaerobic conditions, as many *Endozoicomonadaceae* genomes encode at least one fumarate reductase. However, anaerobic respiration and the role of alternative electron acceptors in *Endozoicomonadaceae*, as well as detailed studies of its nitrate reductases, remain untapped in the literature, highlighting yet another unexplored aspect of this family’s metabolism and ecology.

Steroid-degrading *Gammaproteobacteria* have been isolated from sponges and corals, suggesting bacteria–host symbiosis is influenced by microbial steroid metabolism^89^. In animals, steroid hormones modulate many physiological functions, including development, reproduction, and communication^89^. Steroid hormones serve as a source of carbon and energy for many bacteria, as they are highly reduced carbon-rich compounds derived from cholesterol^89^. Although no genes related to steroid utilization were found in any *Endozoicomonadaceae* genomes^77^ (see Supplementary Data 4–6), *E. acroporae* was experimentally verified to use cholesterol^87^ and *E. montiporae* to use testosterone as a sole carbon source^79^. Under thermal stress, *E. acroporae* converts cholesterol into testosterone and progesterone, two hormones that prime the coral’s immune system and inhibit pathogenic bacteria and fungi (illustrated in Fig. 4)^87^. The ability of *Endozoicomonas* spp. to convert coral steroids may enhance their close association and interkingdom symbiosis^90^.

Genome analyses also suggest that *Endozoicomonadaceae* metabolize diverse carbon sources (Supplementary Data 4–6) and nutritional symbiosis may be complemented by extracellular enzymes that can degrade polysaccharides, such as chitin and cellulose, which are then used by the host^4,76,91^. Filter- and suspension-feeding marine invertebrates process chitin-rich particles from the water column, including phyto- and zooplankton, and host a microbiota capable of hydrolysing chitin and/or utilizing its degradation products^4^. *Endozoicomonadaceae* species are presumably important contributors to chitin turnover, as many *Endozoicomonadaceae* genomes are enriched in chitin degradation-related genes, such as endo-chitinases and chitin-binding proteins^4,91^. They presumably benefit the holobiont by providing energetic sugar derivatives and structure the microbial community through cross-feeding with other symbiotic bacteria (illustrated in Fig. 4)^4^. Chitinases may also protect hosts against fungal infections, as fungi have chitin in their cell walls^49^. Recent studies suggest that chitin oligosaccharides act as chemical cues to attract symbionts in marine symbioses^92,93^. While chitin is a structural component in several marine organisms, including sponges^94^, there is little evidence of its presence in corals, except for black corals. However, several coral species possess chitin synthase genes^95^, indicating that chitin oligosaccharides may play a role in interkingdom signalling, promoting colonization by beneficial symbionts.

Corals are prolific sources of dimethylsulfoniopropionate (DMSP), an organosulfur compound especially abundant in animals that harbour endosymbiotic dinoflagellates^74^. DMSP has antioxidant and osmoprotectant capacities^96^ and is a chemical cue driving bacterial community structuring, thereby affecting host and reef health^97,98^. The majority of DMSP is produced by the Symbiodiniaceae^97,99^, which is then released to the surrounding water, where it is available for microbial catabolism as a source of reduced carbon and sulfur^74^. The genome of *E. acroporae* encodes for a DMSP CoA-transferase/lyase that converts DMSP into dimethylsulphide (DMS), suggesting a role in the coral sulfur cycle (illustrated in Fig. 4)^74^. However, this trait is not universal among *Endozoicomonadaceae* bacteria, and DMSP lyases are only present in some species^77^ (see also Supplementary Data 5).

### Antioxidant and antimicrobial activities

Marine organisms can experience changes in environmental conditions that lead to oxidative stress. Thermal stress, exposure to ultraviolet radiation, and tidal heights cause physiological changes in marine invertebrates, but homoeostasis can be maintained in the holobiont if reactive oxygen species (ROS) are removed^74,100^. *Endozoicomonas* species harbour many oxidative stress-responsive genes, suggesting a role in oxidative stress response^74^. Most *Endozoicomonadaceae* show catalase and oxidase activities (Table 1), allowing them to scavenge hydrogen peroxide and free oxygen radicals (Fig. 4). The genomes of *Candidatus Gorgonimonas* encode rubredoxins that function as electron carriers in various biochemical processes and may play key roles in reducing ROS^4^. In addition to increased catalase, heat-shock protein, and co-chaperone levels, *E. montiporae* expressed high levels of hydroperoxyl fatty acid reductases during mild heat stress, an antioxidant enzyme that removes ROS, likely preventing cellular damage^100^. However, when the temperature exceeded their thermal threshold, *E. montiporae* cells could not cope, and the expression levels of ROS-scavenging enzymes decreased^100^. Moreover, antioxidant activities may be conferred by aryl polyene biosynthesis, while ectoine production may confer osmotic stress protection^35^. Aryl polyene and ectoine BGCs are carried by several *Endozoicomonas* genomes of clades A and B but are absent in all other *Endozoicomonadaceae* lineages (Fig. 1 and Supplementary Data 3). While ROS-scavenging is generally considered a beneficial trait that may support the coral holobiont under oxidative stress, it is important to note that hosts also produce reactive oxygen species as part of their innate immune response to control microbial communities or ward off invaders. Thus, ROS-neutralizing mechanisms in *Endozoicomonadaceae* may enable them to persist under conditions that suppress other microorganisms. Whether this trait is ultimately beneficial or detrimental to the host likely depends on the specific context and remains an important area for further investigation.

Antimicrobial defence is crucial for sessile marine invertebrates such as sponges, corals, anemones, and tunicates, where *Endozoicomonadaceae* spp. abound, especially when subjected to abiotic stresses that can induce microbial shifts and facilitate pathogen invasion. *Candidatus* Gorgonimonas, *E. montiporae*, and *Endozoicomonas* sp. ONNA2 do not harbour any secondary metabolite encoding BGCs (Fig. 1 and Supplementary Data 3), suggesting that these symbionts may not be involved in the chemical defence of their host^4^. Other *Endozoicomonadaceae* genomes carry BGCs which could be involved in the production of compounds with antimicrobial activities, for example, ribosomally synthesized and post-translationally modified peptide products (RiPPs), NRPS, beta-lactones, and T3PKS^35^, the latter being a signature of *Kistimonas* and *Parendozoicomonas*, apart from *P. verongulae* (Fig. 1 and Supplementary Data 3). Yet in contrast to the closely related *Zooshikellaceae* family, which has rich BGC profiles, *Endozoicomonadaceae* BGC profiles appear limited. Moreover, experimental evidence for any ecologically relevant inhibitory activity of *Endozoicomonadaceae* spp. against marine pathogens is lacking. *Endozoicomonadaceae* spp. may keep pathogens in check by other means, using chitinases, steroids, and siderophores, as discussed above, and by competing with invading microbes for nutrients^101^.

## Outlook and directions for future research

### *Endozoicomonadaceae* for microbiome engineering and as potential probiotics

Mass coral bleaching events resulting from high seawater temperatures are the main causative agent of global reef loss, aggravated by local impacts such as sewage, overfishing, and disease outbreaks^102^. Probiotics are one of the solutions with which scientists are trying to reverse or halt current trends, since, besides boosting host health, they can also enhance stress resilience of endangered organisms^103^. Probiotics are live microorganisms that, when administered in adequate quantity can confer health benefits to the host^104^. Coral probiotics, or beneficial microorganisms for corals (BMCs), have the potential to promote growth rates, decrease their mortality after thermal stress, as well as decrease their stress responses when exposed to combined heat and pathogen loads or even after exposure to a simulated oil spill^103,105^. Their beneficial mechanisms encompass (i) promoting nutrition and growth (e.g., photosynthesis, nitrogen fixation, organic nitrogen and carbon cycling and regulation, DMSP breakdown, siderophore-, B-vitamins and/or urease production); (ii) alleviating stress and the effects of toxic compounds (antioxidants production e.g., catalases, superoxide dismutases, mycosporine-like amino acids, carotenoids; compatible solutes synthesis e.g., betaines, ectoine; toxic compounds breakdown e.g., oil hydrocarbons); (iii) preventing pathogen entry (e.g., production of antibiotics, quorum quenching); and (iv) supporting early-life stage development (e.g., production of signals for larval settlement) (reviewed in Peixoto et al., 2017^106^ and 2021^102^, and Garcias-Bonet et al., 2024^103^). To be considered a BMC, the microorganism must show at least one of these functions^106^.

As detailed in this review, several *Endozoicomonadaceae* species seemingly fulfil many of the above-described beneficial roles, such as their participation in carbon, nitrogen, and sulfur cycling, iron sequestration, alleviation of osmotic and/or oxidative stress through osmolytes, catalases and other antioxidant molecules. Thus, certain *Endozoicomonadaceae* species emerge as potential candidates for coral-probiotic approaches aimed at improving coral fitness and growth or stress resilience. However, while there are multiple examples of the successful application of probiotics in corals^105,107,108^, there are so far no published records of such experiments with *Endozoicomonadaceae*. This is rather surprising, given the large research interest in this group and their prevalence in marine animals and reef ecosystems. Partially, it may be explained by an apparent difficulty in growing *Endozoicomonadaceae* cultures in the laboratory to the high cell densities needed for meaningful in vivo studies with repeated inoculations. Moreover, some *Endozoicomonadaceae* lineages are recalcitrant to cultivation and long-term preservation in the laboratory, making the design of proof-of-concept studies to examine host beneficial traits challenging and hindering our understanding of their potential applicability as live probiotics for marine animals. Yet probiotics developed for the hexacoral *Pocillopora damicornis* were entirely made of *Gammaproteobacteria* such as *Pseudoalteromonas* sp., *Halomonas* sp., and *Cobetia* sp., the latter two belonging, like *Endozoicomonadaceae*, to the order *Oceanospirillales*^105,107^. The successful use of phylogenetically related bacteria suggests that *Endozoicomonadaceae* spp. could likewise be tested for their suitability as coral probiotics in the future, although thorough environmental risk and safety assessments will have to be undertaken, given that some members of the family can be pathogens to fish, clams, and echinoderms.

### Other open questions in *Endozoicomonadaceae* research

The family *Endozoicomonadaceae* establishes relationships that range from mutualistic to pathogenic. This variation is supported by the family’s functional and metabolic diversity, suggesting that the host group, the bacterial lineage, and the environmental context influence the resulting relationship. Understanding the ecology, phenotype, and functions of *Endozoicomonadaceae* remains a key research focus, with many questions still unanswered: (i) when and how symbiosis is established and interkingdom signalling achieved; (ii) the molecular mechanisms and drivers determining mutualism versus pathogenicity; (iii) the processes by which *Endozoicomonadaceae* shape host microbiomes; and (iv) the influence of environmental conditions, host life history, and host lineage on their abundance and distribution. Multifaceted, hypothesis-driven, experimental approaches, and an integrated host–microbiota multi-omics framework are needed to fully comprehend the role of this widespread symbiotic group. Nyholm and colleagues recently proposed and developed a holo-omics approach, which comprises a set of experiments to obtain multiple omics data from both host and microbiota domains^109^. Establishing such a holo-omics strategy to study the vast spectrum of *Endozoicomonadaceae*—containing holobionts, which include a variety of marine animals, is a challenging yet timely task, bearing wide implications for our current knowledge of symbiosis, evolution, and conservation across marine ecosystems.

While studies of the diversity and function of symbiotic microbiomes have been greatly spurred by advances in next-generation sequencing technologies, the need to illuminate the genome architecture and coding potential from the host side remains to be fully addressed. Initiatives such as the Aquatic Symbiosis Genome Project, which aims at delivering resolved hologenomes (that is, the host’s genome plus the genomes of its associated microbiome) across hundreds of freshwater and marine symbioses, hold promise in augmenting our views of the molecular mechanisms involved in the establishment and maintenance of these relationships.

### Deciphering phage–host relationships in the *Endozoicomonadaceae* family

We foresee that our increasing ability to sort and decipher viromes from complex symbioses will foster the identification of so-far unknown *Endozoicomonadaceae*-bacteriophage couplings, aiding in the delineation of the evolutionary trajectories of these bacteria across the symbiosis spectrum. This knowledge, coupled with an improved cultivability of *Endozoicomonadaceae* spp. and their bacteriophages, may foster future phage therapy endeavours to regulate the dynamics of pathogenic *Endozoicomonadaceae*, especially in a global warming scenario in which pathogen proliferation across coastal and food production ecosystems is anticipated.

Furthermore, bacteriophages have been shown to modulate the expression of ankyrin repeats by bacterial symbionts of marine sponges^110^. Therefore, they likely play a decisive role in shaping microbiome assembly in these hosts, as ankyrin repeats are acknowledged to enable sponge symbionts to evade phagocytosis^111^. However, while these ELPs are thought to play a role in host interactions and genome evolution, the specific mechanisms underlying their contribution to symbiosis remain largely unresolved. It is likely that analogous mechanisms take place across different symbiotic systems, yet the extent to which phage-mediated gene expression governs the evolution and population dynamics of *Endozoicomonadaceae* within their hosts remains to be elucidated.

### Methodological approaches to resolve the ecology of *Endozoicomonadaceae*

Understanding the ecology of *Endozoicomonadaceae* can also benefit from stable isotope probing (SIP) to determine the fate of certain metabolic compounds and reveal the cross-feeding mechanisms and food webs within the holobiont^112^. Secondary ion mass spectrometry (SIMS)^112^ could provide further information regarding *Endozoicomonadaceae* location within the host and quantify their metabolic activity. This could reveal how metabolites are shared with the host, as well as account for single-cell heterogeneity, a phenomenon of particular importance when hosts harbour multiple bacterial species or genotypes^1,17^. Nanoscale SIMS (NanoSIMS) has the potential to overcome SIP and is useful to detect molecules that are consumed and produced by *Endozoicomonadaceae* aggregates^113^ and can be combined with FISH (FISH-NanoSIMS) to reveal the elemental distributions between the *Endozoicomonadaceae* aggregates, the host, and other symbiotic members^73^.

In addition, genetic manipulation of *Endozoicomonadaceae* isolates could be tried to produce strains (through gene knock-ins and knock-outs) that enable scientists to explore various aspects of molecular interactions^114^, such as understanding the function of certain proteins and pathways involved in nutrient cycling, stress responses and symbiosis establishment within the host^115^. These approaches hold the potential to unveil the overall dynamics between *Endozoicomonadaceae* and the host, as well as with members of the host microbiome.

## Methods

### Genome selection and classification

Eighty-five publicly available genome assemblies from members of the families *Endozoicomonadaceae* and *Zooshikellaceae* were retrieved from the National Center of Biotechnology Information (NCBI; *N* = 79)^116^, the rapid annotations using subsystems technology (RAST, *N* = 5)^117^, and the integrated microbial genomes (IMG; *N* = 1)^118^. Genomes were uploaded on the Department of Energy (DOE) Systems Biology Knowledgebase (KBase) online platform (www.kbase.us)^119^, where they were assessed for their completeness and contamination with CheckM (v1.0.18)^120^. All genomes had their GC content and genome size retrieved from the respective assembly files on KBase. Detailed information on the databases used for genome retrieval, the assembly accession numbers, and genome quality of all *Endozoicomonadaceae* genomes explored in this study is provided in Supplementary Data 1. Genomes were taxonomically classified with the Genome Taxonomy Database toolkit (GTDB-tk) (v2.3.2)^121^ within KBase (see Supplementary Data 2). To build the final genome dataset for phylogenomic and comparative functional genomic analyses, type strains were preferred over other strains of the same species. Genomes were included based on correct family-level taxonomic assignment and high quality (>70% completeness and <10% contamination), resulting in 54 genomes (50 *Endozoicomonadaceae* and 4 *Zooshikellaceae*) out of the 85 initially retrieved (Supplementary Data 1).

### Phylogenomic analysis

A phylogenomic analysis was performed for the 54 selected genomes, and three available genomes of the family *Hahellaceae* were used as an outgroup to root the tree (*Hahella ganghuensis* DSM 17046 (GCA 000376785.1), *H. chejuensis* KCTC 2396 (GCA 000012985.1), and *Allohahella marinimesophila* H94 (GCA 039538005.1)). The tree was constructed with the SpeciesTreeBuilder (v0.1.4) application using the function ‘Insert Set of Genomes into Species Tree’ of the KBase online platform^119^. The genomes were previously annotated with Prokka (v1.14.5)^122^ and used as input within KBase. Sequences were aligned with a multiple sequence alignment based on a set of 49 core clusters of orthologous groups (COGs) of proteins^119^. The FastTree2 algorithm^123^ was used with the maximum likelihood (ML) method and Jukes–Cantor evolutionary model with a CAT approximation to generate a tree with 1000 repetitions to estimate bootstrap support values^123^.

### Genome functional annotation

Secondary metabolite encoding biosynthetic gene cluster (BGCs) annotation of the 54 genomes was done with the antiSMASH bacterial version 7.0 online platform^124^ (see Supplementary Data 3). The detection strictness was set to ‘relaxed’, and the extra features ‘KnownClusterBlast’, ‘ClusterBlast’, and ‘MIBiG cluster comparison’ were enabled. Genome-wide COG and protein families (Pfam) annotations (Supplementary Data 4, 5) were performed for the 54 selected genomes using the automated genome annotation pipeline ‘Melange’^4,125^, as documented on GitHub (https://github.com/sandragodinhosilva/melange). Supplementary Data 6 summarizes the specific COG and Pfam functions selected for this review.

### Reporting summary

Further information on research design is available in the Nature Portfolio Reporting Summary linked to this article.

## Supplementary information

Description of Additional Supplementary Files

Supplementary Data1-6

Reporting Summary

## Data Availability

The authors confirm that all supporting data of the genomic analyses have been provided within the article. All genome assemblies referenced in the manuscript are publicly available from established databases (NCBI, RAST and IMG) and the respective accession numbers have been provided for each genome both in Fig. 1 and in the supplementary material.
