# Supplementary material for: A genomic view of the bacterial family Endozoicomonadaceae in marine symbioses
Source: Commun Biol. 2025 Oct 2;8:1418. doi: 10.1038/s42003-025-08828-9 (PMC12491435; doi:10.1038/s42003-025-08828-9)
Supplement: Supplementary file 1 — Description of Additional Supplementary Files [file 42003_2025_8828_MOESM1_ESM.pdf]

Description of Additional Supplementary Files

File name: Supplementary Data

Supplementary Data 1
Description: List of published strains and species of the families *Endozoicomonadaceae* and *Zooshikellaceae* (*N*=88 strains).

Supplementary Data 2
Description: GTDB-tk classification results (*N*=85 genomes).

Supplementary Data 3
Description: Secondary metabolite encoding biosynthetic gene clusters (BGCs) annotation of the *Endozoicomonadaceae* (*N*=50) and *Zooshikellaceae* (*N*=4) genomes using antiSMASH bacterial version 7.0.

Supplementary Data 4
Description: Cluster of Orthologous Groups (COGs) of proteins annotation of the *Endozoicomonadaceae* (*N*=50) and *Zooshikellaceae* (*N*=4) genomes.

Supplementary Data 5
Description: Protein families (Pfam) annotation of the *Endozoicomonadaceae* (*N*=50) and *Zooshikellaceae* (*N*=4) genomes.

Supplementary Data 6
Description: Counts of selected functional features annotated in the *Endozoicomonadaceae* genomes (*N*=50).
